# High-Performance Thin Film Transistor with an Neodymium-Doped Indium Zinc Oxide/Al_2_O_3_ Nanolaminate Structure Processed at Room Temperature

**DOI:** 10.3390/ma11101871

**Published:** 2018-10-01

**Authors:** Rihui Yao, Xiaoqing Li, Zeke Zheng, Xiaochen Zhang, Mei Xiong, Song Xiao, Honglong Ning, Xiaofeng Wang, Yuxiang Wu, Junbiao Peng

**Affiliations:** 1 Institute of Polymer Optoelectronic Materials and Devices, State Key Laboratory of Luminescent Materials and Devices, South China University of Technology, Guangzhou 510640, China; yaorihui@scut.edu.cn (R.Y.); 18826447009@163.com (X.L.); 201520114219@mail.scut.edu.cn (Z.Z.); zhangxc_scut@foxmail.com (X.Z.); xiaochanglang@163.com (M.X.); xiaosong@tcl.com (S.X.); psjbpeng@scut.edu.cn (J.P.); 2 Shenzhen China Star Optoelectronics Technology Co., Ltd (CSOT), Shenzhen 518132, China; 3 Institute of Semiconductors, Chinese Academy of Science, Beijing 100083, China; wangxiaofeng@semi.ac.cn; 4 School of Automation Science and Engineering, South China University of Technology, Guangzhou 510640, China

**Keywords:** thin film transistor, Nd:IZO/Al_2_O_3_ nanolaminate structure, room temperature

## Abstract

In this work, a high-performance thin film transistor with an neodymium-doped indium zinc oxide (Nd:IZO) semiconductor via a room temperature approach and adopting the Nd:IZO/Al_2_O_3_ nanolaminate structure was investigated. The effects of the ultrathin Al_2_O_3_ layer and the thickness of Nd:IZO layer in the nanolaminate structure on the improvement of electrical performance and stability of thin film transistors (TFTs) were systematically studied. Besides the carrier movement confined along the near-channel region, driven by the Al_2_O_3_ layer under an electrical field, the high performance of the TFT is also attributed to the high quality of the 8-nm-thick Nd:IZO layer and the corresponding optimal Nd:IZO/Al_2_O_3_ interface, which reduce the scattering effect and charge trapping with strong M–O bonds in bulk and the back-channel surface of Nd:IZO, according to the X-ray reflectivity (XRR), X-ray photoelectron spectroscopy (XPS), and micro-wave photo conductivity decay (μ-PCD) results. As a result, the Nd:IZO/Al_2_O_3_ TFT exhibits an outstanding performance, with a high *μ*_sat_ of 32.7 cm^2^·V^−1^·s^−1^, an I_on_/I_off_ of 1.9 × 10^8^, and a low subthreshold swing (*SS*) value of 0.33 V·dec^−1^, which shows great potential for the room temperature fabrication of TFTs in high-resolution or high-frame-rate displays by a scalable, simple, and feasible approach.

## 1. Introduction

Great interest in metal oxide semiconductors (MOS) for thin film transistors (TFTs) has grown dramatically in recent years, due to their visible-light transparency, good uniformity, compatibility with different substrates, fewer limitations on processing temperature, and the high mobility to drive flexible Active Matrix Organic Light Emitting Diode (AMOLEDs) or high-resolution displays [1,2]. As the most widely-studied MOS material in TFTs, indium gallium zinc oxide (IGZO), the saturation mobility of which is usually 10~20 cm^2^·V^−1^·s^−1^ [3,4], is limited by its natural structure, especially when the processing temperature is strictly controlled for some device applications on flexible plastic substrates or nanopapers. As reported by our previous works [5,6], we have successfully promoted the mobility of a-IGZO TFTs to 25 cm^2^·V^−1^·s^−1^ under room temperature conditions by using an a-IGZO/Al_2_O_3_ nanolaminate structure. However, it is difficult to have a higher breakthrough on device performance for the a-IGZO TFT, which is still not sufficient for the high-resolution or high-framerate displays requiring mobility of over 30 cm^2^·V^−1^·s^−1^ [7]. Therefore, efforts to improve the performance of MOS TFTs by using neodymium-doped indium zinc oxide (Nd:IZO) will be demonstrated in this research. Compared to a Ga–O (354 kJ/mol) bond [8], the bonding strength of Nd–O (703 kJ/mol) [9] is much stronger, so it is more efficient to suppress the formation of oxygen vacancy-related defects and improve the mobility of carriers. In addition, the electronegativity of Nd is as low as 1.1, so the carrier concentration can be controlled. In this work, we demonstrate a high-performance TFT (mobility > 30 cm^2^·V^−1^·s^−1^) with an Nd:IZO semiconductor processed by a room temperature approach, and investigate the effects of a sputtered Nd:IZO/Al_2_O_3_ nanolaminate structure. Moreover, the thickness of the Nd:IZO layer in Nd:IZO/Al_2_O_3_ structure also showed a great impact on the device performance, due to the nature of film growth during the sputtering process.

## 2. Materials and Methods

**Figure 1:**
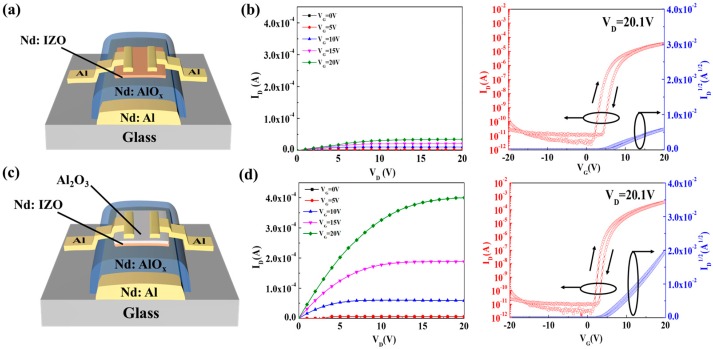
The schematic diagrams and output/transfer characteristics of (**a**,**b**) a single neodymium-doped indium zinc oxide (Nd:IZO) thin film transistor (TFT) and (**c**,**d**) an Nd:IZO/Al_2_O_3_ TFT.

Figure 1a,c shows the schematic diagrams of a single Nd:IZO TFT and an Nd:IZO/Al_2_O_3_ TFT, respectively. Besides the insertion of an ultrathin Al_2_O_3_ layer with a thickness of 3 nm for the Nd:IZO/Al_2_O_3_ TFT, the processes for the fabrication of both devices are consistent. Firstly, a 300-nm-thick Nd-doped aluminum was deposited as a gate metal on glass by direct-current (DC) magnetron sputtering and patterned by photolithography. Then, a 200-nm-thick Nd:AlO*_x_* was formed on the surface of the gate metal by anodic oxidation. The introduction of the Nd element was also reported to contribute to a smooth surface of the Al electrode and high dielectric properties of the AlO*_x_* gate insulator [10]. The Nd:IZO semiconductor layer was deposited by radio-frequency (RF) magnetron sputtering with a power of 60 W and a pressure of 3 mTorr in a mixed atmosphere (Ar:O_2_ = 20:1) using the Nd:IZO target with an Nd_2_O_3_:In_2_O_3_:ZnO ratio of 1:62.5:36.5 wt.%. For the Nd:IZO/Al_2_O_3_ TFT, the 3-nm ultrathin Al_2_O_3_ layer was then prepared by RF magnetron sputtering, with a power of 120 W and a pure Ar pressure of 1 mTorr onto the surface of the Nd:IZO layer for 120 s. Finally, the 150-nm-thick Al source/drain (S/D) electrodes were prepared by DC magnetron sputtering and patterned by a shadow mask. The width/length ratio (W/L) of all devices was 500/100 μm·μm^−1^. Besides the baking process for the photoresist, carried out on thermal platform under 150 °C for 1 min, no further thermal treatment was adopted during the whole process. The electrical characteristics of the TFTs were measured by a semiconductor parameter analyzer (Agilent 4155 C, Santa Clara, CA, USA) under ambient conditions. X-ray photoelectron spectroscopy (XPS) analysis was carried out to investigate the chemical changes in the oxide films by using a THERMO ESCALAB250Xi (Thermo Fisher Scientific, Waltham, MA, USA) with an Al Ka (hν = 1486.6 eV) 15 kW beam spot source. The thickness (±0.1 nm), roughness and density of the Nd:IZO films were determined by X-ray reflectivity measurements (XRR; PANalytical EMPYREAN, Almelo, The Netherlands) using a Cu-Ka X-ray source at 40 kV and 40 mA. Microwave photo conductivity decay (μ-PCD; KOBELCO, Kobe, Japan) was used to characterize the relative carrier concentrations of the Nd:IZO/Al_2_O_3_ films and their relationship with the electrical performance of the TFT devices.

## 3. Results and Discussion

Figure 1b,d shows the output and transfer characteristics of the single Nd:IZO TFT and the Nd:IZO/Al_2_O_3_ TFT—therein, the thicknesses of Nd:IZO layers are both 8 nm. The output characteristics of TFTs were obtained with V_G_ = 0~20 V in steps of 5 V, and the transfer curves were measured with V_D_ = 20.1 V (saturation regime). The single Nd:IZO TFT could already exhibit an electrical performance with a saturation mobility (*μ*_sat_) of 4.1 cm^2^·V^−1^·s^−1^ and an on-to-off current ratio (I_on_/I_off_) of 1.0 × 10^7^, which implies an acceptable concentration of carriers for the room-temperature-prepared Nd:IZO layer. However, the mobility and on-state current (I_on_) is still low, due to the high density of defects in the as-deposited oxide thin film, especially near the back-channel region. By inserting an ultrathin Al_2_O_3_ film upon the Nd:IZO layer, as shown in Figure 1d, an obvious enhancement on the I_on_ can be observed, the corresponding saturation mobility of the Nd:IZO/Al_2_O_3_ TFT can be remarkably improved to 32.7 cm^2^·V^−1^·s^−1^, and the I_on_/I_off_ reaches 1.9 × 10^8^. Moreover, the hysteretic phenomenon reduces in the transfer curves of Nd:IZO/Al_2_O_3_ TFT. According to our previous study [11] and other reports [12,13], the Al_2_O_3_ acts as an electrical controller layer, inducing high-flux electron movement in the bulk and near-channel regions of the semiconductor layer by the carrier confinement effect under an electrical field, which avoids the scatting and trapping effect by the back-channel defects, and hence efficiently improves the device’s mobility. Meanwhile, in the Nd:IZO/Al_2_O_3_ system, the Al_2_O_3_ layer should have a specific role in the modification of the Nd:IZO semiconductor layer, which will be further discussed in this paper.

### 3.1. Effect of the Ultrathin Al_2_O_3_ Layer

#### 3.1.1. Chemical Structure

**Figure 2:**
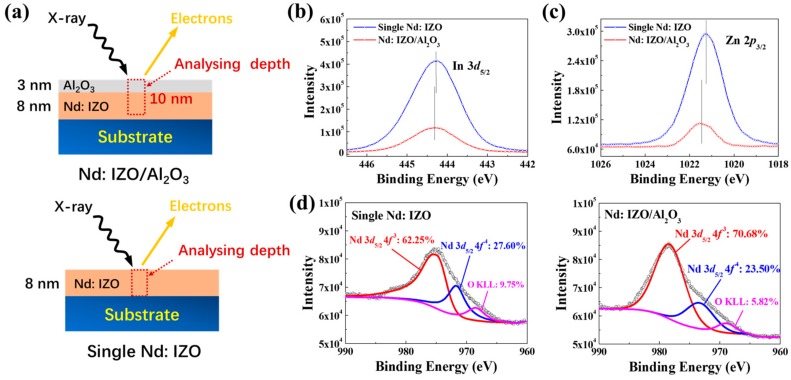
The (**a**) schematic illustration and (**b**) In 3*d*_5/2_, (**c**) Zn 2*p*_3/2_, and (**d**) Nd 3*d*_5/2_ core level spectra of the single Nd:IZO film and the Nd:IZO/Al_2_O_3_ stacked films.

To further investigate the effect of ultrathin Al_2_O_3_ on Nd:IZO layer, X-ray photoelectron spectra (XPS) measurements on the Nd:IZO/Al_2_O_3_ (8 nm/3 nm) multilayered films and a single Nd:IZO film (8 nm) were carried out. As shown in Figure 2a, the analysing depth for an XPS measurement on oxide films can reach ~10 nm, thus allowing chemical analysis for the Nd:IZO film covered by a 3-nm ultrathin Al_2_O_3_ layer. Herein, it is difficult to judge the difference between oxygen vacancies in the Nd:IZO by the O 1*s* core level spectra, because of the existence of Al_2_O_3_. Therefore, the XPS results for In 3*d*_5/2_, Zn 2*p*_3/2_, and Nd 3*d*_5/2_ core level spectra of the both samples were acquired as shown in Figure 2b–d. Compared to the single Nd:IZO film, all of the peaks of Nd:IZO with Al_2_O_3_ exhibit the positive shifts towards higher energy direction, indicating that the introduction of the Al_2_O_3_ layer can prevent oxygen decomposing from the metal–oxygen (M–O) bonds near the back-channel surface of the Nd:IZO film, which reduces the density of defects and thus enhances carrier mobility. Moreover, the decomposition of the Nd 3*d*_5/2_ lines through nearly Gaussian fitting shows that the Nd:IZO film covered by Al_2_O_3_ layer has a lower content of the Nd 3*d*_5/2_4*f*^4^ configuration, which is considered to be associated with scattering centers or charge traps [14,15].

#### 3.1.2. Electrical Stabilities

**Figure 3:**
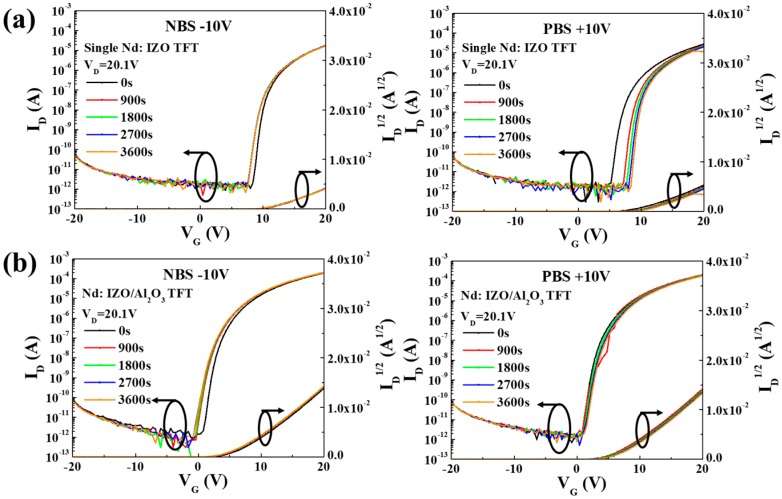
The electrical stabilities of (**a**) a single Nd:IZO TFT and (**b**) a Nd:IZO/Al_2_O_3_ TFT under NBS (V_G_ = −10 V) and PBS (V_G_ = +10 V) for 1 h.

Figure 3a,b show the electrical stabilities of a single Nd:IZO TFT and a Nd:IZO/Al_2_O_3_ TFT under negative/positive bias stress (NBS/PBS) in 1 h, respectively. Both of the TFTs exhibit favorable stabilities under NBS after the first 900 s, with slight shifts of V_on_, indicating that the introduction of the Nd element can actually prevent the ionized oxygen vacancies from migrating to the semiconductor–insulator interface under the negative bias field [16,17]. However, the PBS result with a positive shift of V_on_ for the single Nd:IZO TFT shows that the diffusion of absorbed water or oxygen molecules can efficiently affect the device stability [18], due to the existence of back-channel defects in the sputtered Nd:IZO layer; with a thickness of only 8 nm, the △V_on_ can reach 3.2 V after 1 h under PBS. Therefore, it is essential to introduce an ultrathin Al_2_O_3_ film, which also acts as a good passivation layer. As a result, the Nd:IZO/Al_2_O_3_ TFT exhibits an outstanding stability under PBS, so that no obvious change is observed on the device transfer characteristics.

### 3.2. Effect of the Thickness of the Nd:IZO Layer in an Nd:IZO/Al_2_O_3_ Structure

#### 3.2.1. Device Performance and Film Qualities

**Figure 4:**
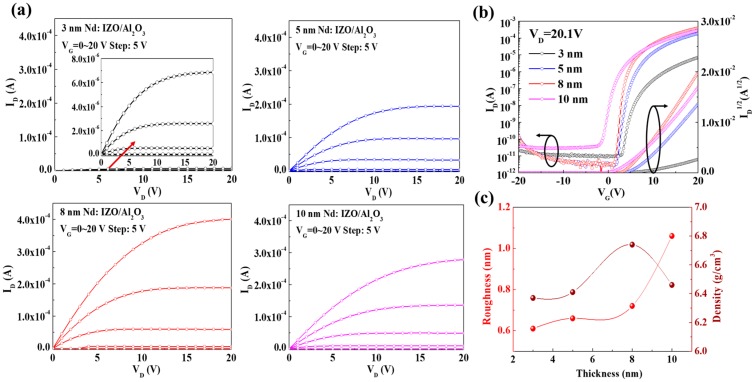
The (**a**) output and (**b**) transfer characteristics of the Nd:IZO/Al_2_O_3_ TFTs with different thicknesses of the Nd:IZO layers; (**c**) the film density and roughness for the corresponding Nd:IZO layers.

Figure 4a,b shows the output and transfer characteristics of the Nd:IZO/Al_2_O_3_ TFTs with different thickness (3, 5, 8, and 10 nm) of the Nd:IZO layers, and the corresponding electrical parameters are summarized in Table 1. The thickness of the Nd:IZO layer in an Nd:IZO/Al_2_O_3_ structure shows a great impact on the device performance. A significant trend can be found that the device performance increased at first, but then degraded as the Nd:IZO thickness increased from 3 to 10 nm. As a result, the TFT with an Nd:IZO thickness of 8 nm exhibits the best electrical properties, with an I_on_ of 4.5 × 10^−4^ A, a *μ*_sat_ of 32.7 cm^2^·V^−1^·s^−1^, an I_on_/I_off_ of 1.9 × 10^8^, and an *SS* value of 0.33 V·dec^−1^. The XRR measurements on the 3, 5, 8, and 10-nm-thick Nd:IZO films were adopted, and the information for film density and roughness was acquired, as shown in Figure 4c. The density of Nd:IZO reaches 6.74 g·cm^−3^, with the optimal thickness of 8 nm, which contributes to fewer structural defects and consequently a higher device performance. Meanwhile, as the thickness increases, the roughness of the Nd:IZO increases continuously, and reaches 1.2 nm when the thickness is 10 nm, which is due to the nature of film growth during the sputtering process. The Nd:IZO/Al_2_O_3_ interfaces are sensitive to the surface roughness of over 1 nm for the Nd:IZO layers, since the thickness of upper Al_2_O_3_ layers are only 3 nm. Therefore, the rough back-channel surface of the Nd:IZO layer would enhance the carrier scattering effect and deteriorate the interface between Nd:IZO and Al_2_O_3_, consequently weakening the electrical properties of the Nd:IZO/Al_2_O_3_ TFTs.

#### 3.2.2. The Nd:IZO/Al_2_O_3_ Interfaces

**Figure 5:**
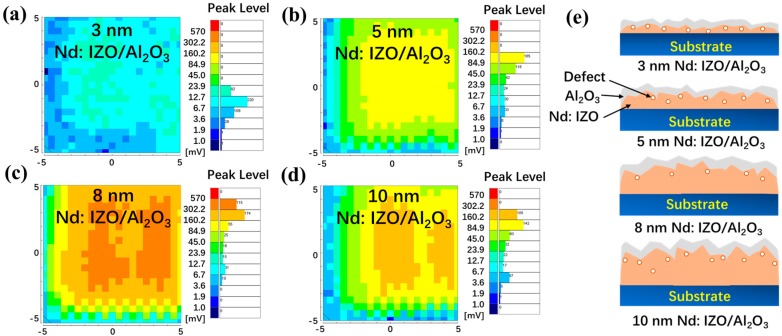
The microwave photo conductivity decay (μ-PCD) peak value mapping scan results for the Nd:IZO/Al_2_O_3_ stacks with an Nd:IZO thickness of (**a**) 3 nm, (**b**) 5 nm, (**c**) 8 nm, and (**d**) 10 nm; and (**e**) the schematic diagrams for the growth process of these films.

Microwave photoconductivity decay (μ-PCD) mapping scan measurements were carried out for the above-mentioned Nd:IZO/Al_2_O_3_ stacks on glass with a size of 10 × 10 mm^2^, in order to further investigate the effect of Nd:IZO thickness on the Nd:IZO/Al_2_O_3_ interfaces, as shown in Figure 5a–d. The Nd:IZO/Al_2_O_3_ stacks with an Nd:IZO thickness of 8 nm exhibit the highest peak mean value of the μ-PCD signal, which indicates the highest relative concentration and mobility of carriers, as well as the lowest density of shallow localized defects [19,20,21] that lead to the best electrical performance of the Nd:IZO/Al_2_O_3_ TFT. Associated with the XRR results, the growth of the Nd:IZO film during sputtering process can be simply demonstrated by Figure 5e. As the initial state of formation for continuous film, the 3-nm-thick Nd:IZO film with high defect density and low carrier concentration results in the poor performance of the TFT device. With the increase of sputtering time and film thickness, the Nd:IZO films become dense and the number of conductive carriers increases, so that the current driving capability of the corresponding TFTs are enhanced. However, as mentioned above, the roughness of the Nd:IZO increases as well, which leads to an increase of the back-channel defects in the Nd:IZO layer and thus deteriorates the Nd:IZO/Al_2_O_3_ interface, according to the μ-PCD results, reducing the mobility of the carriers by scattering and trapping effects.

#### 3.2.3. Effect of Neodymium Concentrations

**Figure 6:**
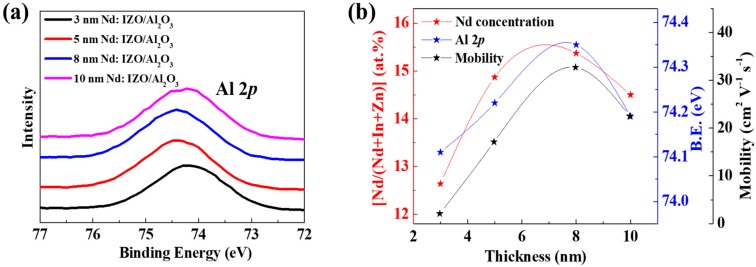
The (**a**) Al 2*p* core level spectra of the Nd:IZO/Al_2_O_3_ stacked films with different Nd:IZO thickness, and (**b**) its relationship with the Nd concentrations in Nd:IZO films and corresponding device mobility.

Furthermore, the XPS measurements on the Nd:IZO/Al_2_O_3_ stacks can also unveil the effect of varying Nd:IZO thicknesses. As shown in Figure 6a,b, a good agreement between variations in the binding energy of the Al 2*p* peak and the ratio of (Nd/(Nd + In + Zn) at.%) implies that the higher concentration of Nd can also contribute to a stronger formation of Al–O bonds (reflected by the positive shift of the Al 2*p* peak) [22], forming a tighter interface between Nd:IZO and Al_2_O_3_. The variation in Nd concentrations of the Nd:IZO films with varying thickness can be attributed to the different depositing rate of Nd/In/Zn atoms during the sputtering process. As the sputtering proceeds, the atomic ratio of each element should first gradually change, and then become stable as soon as the film reaches a certain thickness. According to the XRR/μ-PCD/XPS results above, the thickness of 8 nm is optimal for the high quality of the Nd:IZO film and a favorable Nd:IZO/Al_2_O_3_ interface, leading to the high carrier mobility and consequently, the high electrical performance of Nd:IZO/Al_2_O_3_ TFT.

## 4. Conclusions

In conclusion, we demonstrated a high-performance TFT with an Nd:IZO semiconductor via a room temperature approach, by adopting an Nd:IZO/Al_2_O_3_ nanolaminate structure. The Al_2_O_3_ layer is considered to induce high-flux electron movement in the bulk and near-channel region of the Nd:IZO layer, prevent oxygen decomposing from the M–O bonds near the back-channel surface of Nd:IZO, and act as a good passivation layer, so that the Nd:IZO/Al_2_O_3_ TFT remains highly stable under both NBS and PBS. Meanwhile, the thickness of the Nd:IZO layer has a great impact on the electrical performance of Nd:IZO/Al_2_O_3_ TFTs, due to the nature of the sputtering method. The film quality and carrier concentration are optimal when the thickness of the Nd:IZO layer reaches 8 nm, and the high concentration of Nd also benefits the formation of a tight Nd:IZO/Al_2_O_3_ interface with strong Al–O bonds, reducing the scattering centers and charge traps near the back-channel surface. As a result, the Nd:IZO/Al_2_O_3_ TFT exhibits an outstanding performance with a high *μ*_sat_ of 32.7 cm^2^·V^−1^·s^−1^, an I_on_/I_off_ of 1.9 × 10^8^, and a low *SS* value of 0.33 V·dec^−1^, which shows great potential for the room temperature fabrication of TFTs in high-resolution or high-framerate displays by a scalable, simple, and feasible approach.

## Figures and Tables

**Table 1:** A summary of the parameters obtained by the results of electrical measurements and chemical analysis for the Nd:IZO/Al_2_O_3_ films and their corresponding TFT devices.

TFTs/Films	3 nm Nd:IZO/Al_2_O_3_	5 nm Nd:IZO/Al_2_O_3_	8 nm Nd:IZO/Al_2_O_3_	10 nm Nd:IZO/Al_2_O_3_
*μ*_sat_ (cm^2^·V^−1^·s^−1^)	2.1	17.1	32.7	22.5
I_on_/I_off_	2.7 × 10^6^	8.4 × 10^7^	1.9 × 10^8^	5.6 × 10^6^
SS (V·dec^−1^)	0.32	0.41	0.33	0.63
V_on_ (V)	1.6	1.8	1.0	−2.9
Nd:IZO density (g·cm^−3^)	6.37	6.41	6.74	6.46
Nd:IZO roughness (nm)	0.61	0.66	0.72	1.06
μ-PCD peak mean (mV)	9.0	65.6	205.6	97.4
Nd/[Nd + In + Zn] (at.%)	12.64	14.87	15.37	14.50
Al 2*p* E_b_ (eV)	74.11	74.22	74.35	74.19
